# Osteoporosis and HIV Infection

**DOI:** 10.1007/s00223-022-00946-4

**Published:** 2022-01-30

**Authors:** Emmanuel Biver

**Affiliations:** Division of Bone Diseases, Geneva University Hospitals and Faculty of Medicine, University of Geneva, 4 Rue Gabrielle Perret-Gentil, 1205 Geneva, Switzerland

**Keywords:** Osteoporosis, Fracture, Bone microstructure, HIV, Antiretroviral therapy

## Abstract

Life expectancy of people living with HIV (PLWH) is now close to that of the HIV-uninfected population. As a result, age-related comorbidities, including osteoporosis, are increasing in PLWH. This narrative review describes the epidemiology of bone fragility in PLWH, changes of bone features over the course of HIV infection and their determinants, as well as the available evidence regarding the management of osteoporosis in PLWH. The risk of fracture is higher and increases about 10 years earlier compared to the general population. The classical risk factors of bone fragility are very widespread and are major determinants of bone health in this population. The majority of bone loss occurs during virus replication and during immune reconstitution at antiretroviral therapies (ART) initiation, which both increase osteoclast activity. Abnormalities in bone formation and mineralization have also been shown in histomorphometric studies in untreated PLWH. Measurement of bone mineral density (BMD) is the first line tool for assessing fracture risk in postmenopausal women, men above 50 years, and other HIV-infected patients with clinical risk factors for osteoporosis. FRAX underestimates fracture probability in PLWH. In case of indication for anti-osteoporotic drug, bisphosphonates remain the reference option. Calcium and vitamin D supplementation should be considered as ART initiation, since it may attenuate bone loss at this stage. Bone-protective ART regimens improve BMD compared to other regimens, but to a lesser extent than bisphosphonate, and without available data on their influence on the incidence of fracture.

## Introduction

With the continuous raising efficacy of antiretroviral therapies (ART) combinations, the characteristics of the HIV population are changing. Life expectancy of people living with HIV (PLWH) is now close to that of the HIV-uninfected population, resulting in PLWH being an aging population, with an increasing proportion of patients over the age of 50, reaching more than 50% in European countries or in the USA [1, 2].Therefore, PLWH are at greater risk of developing age-related non-communicable diseases, including osteoporosis and fractures, and more attention is needed to prevent or treat these comorbidities [3, 4]. Meanwhile, the majority of PLWH now have an undetectable viral load with stable ART. Bone health in PLWH results from the complex interactions between aging, comorbidities and classical risk factors affecting bone fragility and very common in this population, and, to a lesser extent, the well-controlled HIV infection itself. This narrative review describes the epidemiology of bone fragility in PLWH, the changes of bone features over the course of HIV infection and their determinants, as well as the available evidence regarding the management of osteoporosis in PLWH.

## Epidemiology of Bone Fragility in PLWH

Meta-analyses have consistently reported a higher fracture risk in PLWH, with an increased risk of fragility fracture of 35 to 68% compared to the general population (Table 1) [5–11]. This greater risk of fracture occurs with aging of HIV populations and is observed approximately 10 years earlier than in the general population, mainly in middle-aged populations such as in the 40–49 and 50–59 age groups [12–14]. In elderly populations, similar hip fracture rates have been reported in nursing home residents in the United States with and without HIV [15]. The prevalence of vertebral fracture in PLWH varies from 4.1 to 47% depending on the studies, with a pooled estimated prevalence of 22% [10]. Co-infections of HIV with hepatitis C or B are associated with a higher risk of fracture than HIV infection alone [6, 7, 16]. Similarly to the general population, incident fractures are associated an increased risk of all-cause mortality in PLWH, but with decreasing associations, likely reflecting advances in HIV care (post-fracture, age- and sex-adjusted all-cause mortality rates per 100 person-year decreased from 8.5 during 2000–2004 to 1.9 during 2013–2017) [17]. In this study, the factors significantly associated with all-cause mortality in PLWH with fractures were the observation period 2000–2004 versus 2005–2017, cardiovascular diseases, chronic kidney diseases, co-infection with hepatitis C, lung disease or a history of non-AIDS cancer.

**Table 1:** Pooled risk of fractures in meta-analyses of cohorts and case–control studies in PLWH

Meta-analyses	Number of studies	Pooled risk	Any fractures			Fragility fractures		Hip fractures	Vertebral fractures
			HIV-infected vs non-infected	HIV + HCV co-infected vs HIV mono-infected	HIV + HCV co-infected vs non-infected	HIV-infected vs non-infected	HIV + HCV co-infected vs HIV mono-infected	HIV-infected vs non-infected	HIV-infected vs non-infected
Shiau et al. [5]	4/5	IRR	1.58 (1.25, 2.00)	–	–	1.35 (1.10, 1.65)	–	–	–
Dong et al. [6]	6/4/3	IRR	–	1.77 (1.44, 2.18)	2.95 (2.17, 4.01)	–	1.70 (1.18, 2.43)	–	–
O'Neill et al. [7]	5/2	RR	–	1.57 (1.33, 1.86)	2.46 (1.03, 3.88)	–	–	–	–
Ilha et al. [8]	9	OR	–	–	–	–	–	–	2.30 (1.37, 3.85)
Pramukti et al. [9]	7 6	OR IRR	1.91 (1.14, 3.22) 1.50 (1.27, 1.78)	–	–	–	–	–	–
Starup-Linde et al. [10]	9/6/3	RR	1.53 (1.46, 1.61)	–	–	1.51 (1.41, 1.63)	–	4.05 (2.99, 5.49)	–
Chang et al. [11]	17/13/6/6	RR	1.91 (1.46, 2.49)	–	–	1.68 (1.40, 2.01)	–	1.88 (0.99, 3.57)	1.97 (1.22, 3.20)

*IRR* incidence rate ratio, *RR* relative risk, *OR* odd ratio, *HIV* human immunodeficiency virus, *HCV* hepatitis C Virus, *vs* versus

## Bone Characteristics in PLWH

### Bone Mineral Density and Its Changes Over HIV Infection Time-Course

The prevalence and incidence of osteoporosis in PLWH are increased compared to controls, especially from the fifth decade [14, 18]. This has been demonstrated in various populations, including women in rural South Africa [19]. The magnitude of the difference of bone mineral density (BMD) between PLWH and controls has been estimated in a meta-analysis to lower BMD Z-scores of − 0.36 standard deviations (95% CI − 0.39 to − 0.15) at the spine and − 0.31 standard deviations (− 0.46 to − 0.27) at the hip [10, 11]. In children and adolescents (aged 8–16 years) living with HIV in sub-Saharan Africa, substantial deficits in bone mineral content and density have been reported despite the use of ART. The effect of HIV on BMD was most marked in the late stages of puberty, especially in girls, and with the use of tenofovir disoproxil fumarate (TDF) [20].

**Fig. 1:**
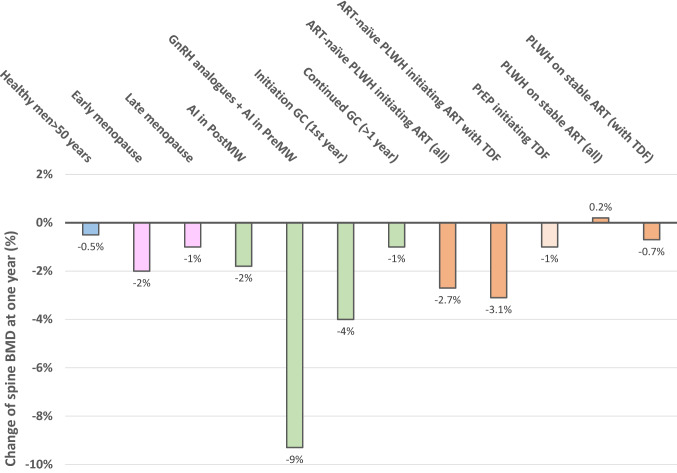
Change of spine BMD after one year in various conditions including HIV. Adapted from Refs. [10, 26–32]. *AI* aromatase inhibitor, *PostMW* postmenopausal women, *PreMW*, premenopausal women, *GnRH* Gonadotropin-Releasing Hormone, *GC* glucocorticoids, *ART* antiretroviral treatment, *PLWH* people living with HIV, *TDF* tenofovir disoproxil fumarate, *PrEP* HIV pre-exposure prophylaxis

However, BMD changes and their magnitude vary over the course of HIV infection [21]. Looking at BMD at the population level in PLWH compared to the uninfected population, BMD is lower even before HIV infection because of the high prevalence of risk factors of osteoporosis in this population (Table 2). Then during untreated HIV infection, BMD decreases due to poor health, weight loss and direct effects of the virus. The greatest decline in BMD is observed after starting ART, for a limited period of 1 to 2 years. Data from the START study clearly demonstrated that bone loss during ART initiation is much greater than that resulting from HIV infection alone [22]. This transient acceleration of bone loss has been attributed to immune reconstitution and is associated with increased bone resorption (via up-regulation of the RANKL/OPG pathway), in addition to the specific effects of some drugs on bone metabolism. Greater decreases in BMD have been reported between baseline and 2 years in PLWH after initiation of TDF-based ART compared to non-TDF-ART [23, 24]. The magnitude of bone loss exceeds that seen after menopause, or approaches that observed during treatment with glucocorticoids or aromatase inhibitors, only within 1–2 years following ART initiation (Fig. 1). Finally, with long-term ART and suppression of viral activity, BMD may increase and then stabilizes. In long-term HIV-positive elderly men aged 60–70 on successful ART for 15 years (median), areal BMD at various bone sites was only 3 to 8% lower than in HIV-negative men matched for age and BMI [25].

**Table 2:** Determinants of osteoporosis and fracture in people living with HIV over the time-course of HIV infection

	Before HIV infection	Untreated HIV infection	ART initiation	Long-term ART-stable PLWH
*Classical risk factors of osteoporosis and fracture* - Non-modifiable: Age, Caucasian ethnicity, prior fractures, parent history of hip fracture - Modifiable: Low BMI, lifestyle: tobacco, alcohol, low physical activity, poor nutrition: low calcium and protein intakes, vitamin D deficiency, hypogonadism in men and early menopause, comorbidities and drugs (glucocorticoids), fall risk	**++**	**++**	**++**	**++**
*Immune and bone cell HIV infection* - Increased osteoclasts differentiation and activity - Decrease osteoblast activity - Pro-adipogenic and inflammatory environment - Immune system modulation	**0**	**++**	**+++**	**0**
*Direct effect of ART* - Renal tubulopathy and urine phosphate wasting (tenofovir) - Interaction with vitamin D metabolism	**0**	**+**	**+**	**+**
*Gut microbial dysbiosis* - HIV-induced gut dysbiosis promoting pro-inflammatory environment - ART effects on gut microbiota	**0/+**	**+++**	**++**	**+**
*BMD changes*	** ↔ \↘**	**↘↘**	**↘↘↘**	** ↔ \↘**

The respective contribution of each determinant block at the population level is indicated (0, no contribution, + low, + + medium, +++ high), and may vary at patient individual levels. Some classical risk factors may be corrected (diet improvement, stop tobacco or alcohol, increase physical activity) while others appears (aging, hypogonadism, comorbidities) in long-term ART-stable PLWH

*HIV* human immunodeficiency virus, *ART* antiretroviral therapy; *PLWH* people living with HIV, *BMD* bone mineral density

### Bone Microstructure

**Fig. 2:**
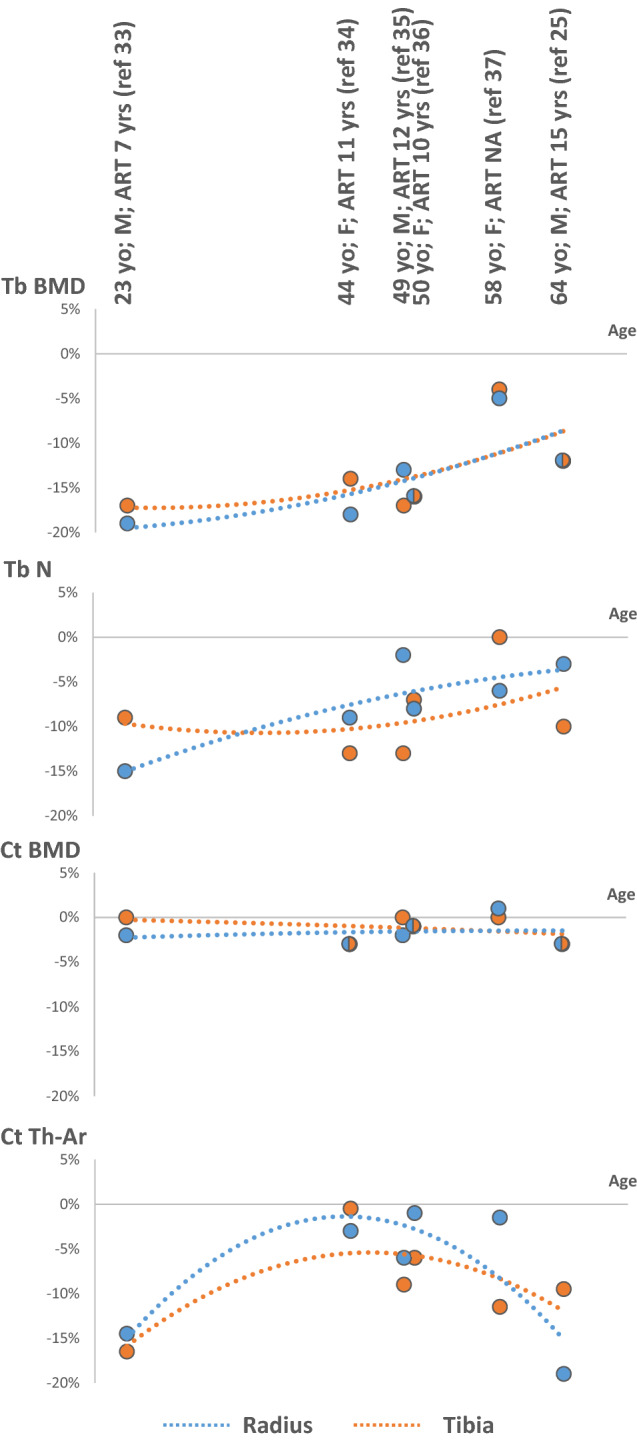
Difference (%) in trabecular and cortical BMD and microarchitecture at the distal radius and tibia between HIV and controls groups in cross-sectional studies, according to age. Sex of the study populations and duration of ART use are indicated (when available). Adapted from Refs. [25, 33–37]. *M* men, *F* women, *ART* antiretroviral therapy, *yo* years old, *yrs* years, *Tb* trabecular, *Ct* cortical, *BMD* bone mineral density, *N* number, *Ar* area, *Th* thickness, *NA* not available

Bone microstructure has also been investigated in PLWH using high-resolution peripheral QCT in several cross-sectional studies, in patients of various age, sex and duration of ART [25, 33–37]. Overall, these data indicate that alterations in volumetric BMD and bone microarchitecture predominate in trabecular rather than cortical bone compartments, except in young and elderly patients in whom defects of cortical thickness or area have also been observed (Fig. 2). The magnitude of the differences in bone traits compared with the respective non-HIV-infected control groups did not exceed 20% at any time and tended to attenuate with aging and duration of ART.

### Bone Histomorphometry

Bone histomorphometry using tetracycline double-labeled transiliac crest biopsies has been reported in two studies: the first one in ART-naïve men and women in the 1990s, including 50% of patients with AIDS-defining opportunistic infections; and a recent one in ART-naïve men who underwent paired biopsies before and 12 months after initiation of TDF/lamivudine/efavirenz (Table 3) [38, 39]. Overall, these data indicate low bone turnover with primarily abnormalities in bone formation and mineralization which are present in untreated PLWH with and without advanced HIV. With ART, there is an increase in bone remodeling but a persistence of the mineralization defect, resulting in an increase in osteoid volume. The decrease in mineralization was not attributable to vitamin D deficiency in these studies. There were no significant change in renal phosphate excretion nor in mineralization parameters with initiation of TDF-containing ART.

**Table 3:** Bone histomorphometry studies using tetracycline double-labeled transiliac crest biopsies in people living with HIV

**Reference**	Serrano S et al., 1995 [38]	Ramalho J et al., 2019 [39]	
*Study characteristics*			
Study population	ART-naïve patients, age 27.9 ± 4.1 years, 59% men, low BMI, 73% intravenous drug abusers, 50% alcohol > 20 g/day, 50% with AIDS-defining opportunistic infections	ART-naïve patients, age 29.6 ± 5.5 years, 100% men, normal BMI (24.7 ± 2.4 kg/m2), 5% alcohol > 30 g/d	
Study years and setting	Before 1995, Barcelona, Spain	2015- 2016, Sao Paulo, Brazil,	
Biopsy timepoint	Before ART initiation	Before ART initiation	12 months after ART initiation
Number of patients	22	20	16
25OH vitamine D (ng/mL)	16.5 ± 9.6	22.0 ± 7.0	27.7 ± 8.7
Parathyroid hormone (pg/mL)	18.7 ± 5.3	31.3 ± 9.2	41.4 ± 12.4
*Static parameters*			
Structural cortical parameters	Not provided	Cortical thickness ↘ in 25%	↗ Cortical thickness by 37%
Structural trabecular parameters	Normal trabecular volume, number and thickness, ↑ trabecular separation	Bone volume/tissue volume ↘ in 20%	No change
Bone formation parameters	↘ Osteoid volume, surface and thickness	Osteoid thickness ↘ in 50%	↗ Osteoid volume (+ 185%, still within the reference range), and osteoblast surface/bone surface (+ 234% increase, to values above the normal ranges)
Bone resorption parameters	↘ Osteoclast number and eroded surface	Osteoclast surface and eroded surface ↗ in 30–40%	↗ osteoclast surface/bone surface (+ 121%, to values above the normal ranges)
*Dynamic parameters*			
Bone formation rate	↘	↘ in 80%	No change
Mineralization	↘	↘ in 60%	No change

*ART* antiretroviral therapy

## Pathophysiology and Risk Factors of Osteoporosis and Bone Fragility in PLWH

Several factors can affect bone fragility and the risk of falls in PLWH, leading to a higher risk of fractures. These factors are linked to the patient himself and to the classical risk factors for fractures which are very common in this population, to factors linked to viral activity and also to the effect of ART [40].

### Contribution of Classical Risk Factors of Bone Fragility

The risk factors of osteoporosis and bone fragility in PLWH are summarized in Table 2. Traditional risk factors include age, low BMI, nutritional factors, toxic habits, as well as hypogonadism. The prevalence of hypogonadism is high in HIV-positive men, approximately 20% of them. It can be primary hypogonadism, but also secondary hypogonadism associated with hypothalamus and pituitary axis dysfunction, obesity, metabolic syndrome or lipodystrophy [41]. In women, HIV infection and menopause are independent predictors of a decrease of BMD [42]. In adults infected with HIV, malnutrition and reduced frequency of mechanical loading activities have been associated with alterations in bone microstructure [43]. The combination of several of these risk factors in patients co-infected with HIV and hepatitis C could explain the particularly increased risk of fractures in this population. Serious falls within the past year, significant enough to warrant a visit to a health care provider, are also, as in the general population, strong predictors of fragility fractures in PLWH on ART [44].

### Virus Activity

Bone loss is accelerated in patients with a high viral load, suggesting a direct effect of virus activity and systemic inflammation on bone metabolism. Recent data demonstrated that HIV has various direct effects on bone cells. HIV affects not only lymphocytes, but also macrophages and osteoclasts via cell-free viruses or by cell-to-cell transfer from infected T-cells. By secreting the receptor activator of nuclear factor kappa-B ligand (RANK-L) and reducing the expression of osteoprotegerin, HIV-infected lymphocytes and macrophages help create a microenvironment that promotes the recruitment of osteoclasts [45]. In addition, in osteoclasts infected with HIV, the expressions of RANKL, tartrate-resistant acidic phosphatase (TRAP) and cathepsin K are increased. These effects, dependent on viral proteins such as Nef or Tat, result in more numerous and more osteolytic osteoclasts having larger and denser sealing zones [46, 47]. HIV also induces early senescence of bone marrow MSCs, the precursors of osteoblasts, and stimulates these cells to secrete inflammatory cytokines such as IL-6 and IL-8. Osteoblast apoptosis is stimulated and the expression of pro-osteoblastic factors such as alkaline phosphatase, runt-related transcription factor 2 (RUNX-2), bone morphogenic proteins (BMP-2, BMP-7) or osteocalcin is decreased. Meanwhile, some viral proteins induce the expression of peroxisome proliferator-activated receptor *γ* (PPARγ). All these factors contribute to a proadipogenic rather than pro-osteogenic phenotype (Table 2) [46].

### Antiretroviral Treatments

ART also affects bone health, independently of the indirect and transient decrease in BMD observed after the start of any ART regimen, associated with immune reconstitution (Table 2) [48, 49]. This has been particularly demonstrated with TDF. A meta-analysis showed that PLWH on stable ART only lose bone with TDF-containing ART (Fig. 1) [10]. Another tenofovir prodrug, tenofovir alafenamide has been developed. Due to specific intracellular activation of the prodrug in infected immune cells, circulating tenofovir concentrations are lower with TAF and a lower BMD decrease is observed with TAF than with TDF. The effect of TDF on bone metabolism is at least partly independent of the context of HIV infection since the decrease of BMD has also been reported, compared to placebo or TAF, in the context of HIV pre-exposure prophylaxis (PrEP) [50, 51]. However, the magnitude of bone loss in these studies appears to be lower than in PLWH (0.8% to 1% BMD loss at the spine after 1 year) (Fig. 1). A meta-analysis of randomized controlled trials evaluating the different impacts of various ART on BMD in PLWH showed that loss of BMD was significantly attenuated with abacavir or TAF compared to TDF. After 96 weeks, spine and hip BMD were significantly less reduced with abacavir compared to TDF by 1.37 percentage point (pp) (95% CI 0.58, 2.15) and 1.40 pp (0.75, 2.05), respectively; with TAF compared to TDF by 1.90 pp (1.65, 2.15) and 2.66 pp (95% CI 2.52 to 2.79), respectively [10]. In virologically supressed PLWH, the decrease in BMD induced by TDF is also attenuated by switching to TAF or to another regimen including abacavir or an integrase inhibitor [52, 53]. In these switch studies, BMD remained stable in control groups that continued TDF-containing ART, confirming the lack of significant effect on BMD of stable TDF-ART regimen in virologically suppressed PLWH, and increased by 1–2% after one year in the switched groups. Whether this BMD gain is transient or continues over time is not established.

TDF is also associated in some patients with proximal renal tubulopathy and urine phosphate wasting, which appear to be related to cumulative exposure to TDF, and may persist even after discontinuation of TDF [54]. The pathophysiology of these tubulopathies remains unclear. In addition, the development of hypophosphatemia and osteomalacia is very rare and bone biopsy data reported earlier in the manuscript did not reveal worsening of mineralization with initiation of TDF [39]. The risk of developing tubulopathy seems to be lower with TAF [55].

### Contribution of Gut Microbiome

Another emerging area in the field of HIV and its impact on bone is the contribution of gut microbial dysbiosis, which affects immune function and HIV persistence (Table 2). Chronic HIV infection induces microbial dysbiosis in the gut, resulting in an overall decrease in microbiome diversity and functional capacity. This dysbiosis leads to an increase of the permeability of the gut barrier which adds to the depletion of T-cells induced by HIV in gut-associated lymphoid tissue, and induces an innate immune activation, resulting in a shift toward a pro-inflammatory cytokine environment with osteoclastogenesis and bone resorption enhancement [56]. In addition to sexual behavior and/or HIV infection, ART also influences the composition of the gut microbiota in PLWH, which changes before and after the start of ART. This has been shown in PLWH and in the context of PrEP and may affect bone health, as demonstrated in the non-HIV population [57–60]. The impact on fecal microbial diversity, which potentially causes intestinal dysbiosis, has been particularly observed in patients receiving ART including nucleotide/nucleoside reverse transcriptase inhibitor (NRTI), with a propensity for intestinal microbiota enriched in Prevotella and poor in Bacteroides [57]. In vitro studies have indicated that zidovudine (AZT), one of NRTI, exhibits antibacterial effects, suggesting potential direct effects on gut microbiota [61]. To what extent targeting gut dysbiosis in ART-stable PLWH could help improve calcium balance and attenuate bone loss remains unexplored.

## Prevention and Management of Osteoporosis in PLWH

### General Preventive and Screening Measures

A periodic assessment of clinical risk factors for bone fragility is recommended in all PLWH, with the implementation of general preventive measures such as the promotion of physical activity, a balanced diet, the cessation of toxic habits when applicable, and prevention of fall in elderly patients.

A DXA scan is recommended for all postmenopausal women, men above 50 years of age, and patients with other clinical risk for fragility fractures, since these patients are more likely to benefit from anti-osteoporotic drugs in case of low BMD [62]. Although the FRAX® tool has been recommended for routine assessment of fracture risk in PLWH over 40 years of age in some guidelines [63], it underestimates fracture risk in PLWH, even including HIV to the set of secondary risks for osteoporosis or after adjustment for the trabecular bone score (TBS). The ratio of observed to predicted fractures is greater than 3 under all of these conditions, possibly because important factors associated with HIV infection are not adequately captured by the tool [64, 65]. Therefore, FRAX should not be considered as a fist-line screening tool for bone fragility in PLWH but may potentially help for decision on intervention with anti-osteoporotic drugs in case of moderately decreased BMD.

### Calcium and Vitamin D

The prevalence of vitamin D insufficiency, i.e. serum 25-OH vitamin *D* < 50 nmol/L (20 ng/mL), is high in PLWH, up to 80% in HIV cohorts [66]. The lastest guidelines of the European AIDS Clinical Society (EACS) recommend checking vitamin D status in PLWH with history of low BMD and/or fracture, high risk for fracture, or with other factors associated with lower vitamin D levels (dark skin, dietary deficiency, avoidance of sun exposure, malabsorption, obesity, chronic kidney disease, and use of efavirenz or protease inhibitors) [67]. A review of 29 clinical studies of vitamin D supplementation in PLWH showed that there is a decrease in inflammation, bone turnover markers, and secondary hyperparathyroidism when vitamin D levels are increased to optimal values regardless of ART [68].

Interventions studies with vitamin D or calcium/vitamin D supplements on bone in PLWH are summarized in Table 4. These studies were performed in children, adolescent or young adults (*n* = 7) or in adults (*n* = 3), with various supplementation regimens regarding the dose and frequency of administration. The equivalent daily doses, calculated according to the doses used in each trial, ranged from approximately 1100 to 7000 IU, thus higher than the daily or equivalent daily dose of 800 UI of vitamin D recommended for maintaining bone health in the elderly and postmenopausal women uninfected with HIV [69]. A decrease of parathyroid hormone (PTH) or bone turnover markers has been observed in some studies [70, 71]. BMD was investigated in 7 studies, with 2 of them showing trends for benefit on BMD in youth [72, 73]. Interestingly, a smaller decrease in hip and spine BMD has been reported, compared to placebo, in ART-naïve adults supplemented with calcium (1000 mg/day) and high-dose of vitamin D (4000 IU/day) at initiation of efavirenz/emtricitabine/TDF [74]. These data are consistent with the pre-existing defect in bone mineralization reported in histomorphometric studies [38, 39]. Since loss of bone mass at ART initiation can be alleviated with vitamin D and calcium supplements, intervention with supplements should be considered early as the initiation of HIV infection management, in case of low calcium dietary intake and low vitamin levels. A specific emphasis on vitamin D in PLWH is important since efavirenz, a non-nucleoside reverse transcriptase inhibitor, has been associated with lower vitamin D levels via a modulation of various cytochromes and enzymes involved in activation or deactivation of vitamin D or vitamin D-binding protein [75, 76]. It is not established whether the optimal vitamin D dosage regimen should differ in PLWH compared to the general population, and the EACS guidelines recommend maintenance with 800 to 2000 IU of vitamin D per day [67]. Vitamin D should be combined with calcium in patients with insufficient dietary calcium intake.

**Table 4:** Interventions studies (randomized controlled trials) with vitamin D or calcium/vitamin D supplements on bone in people living with HIV

Reference	Population	Number	Intervention	Control	Duration	Endpoints	Results
*In children, adolescent and young adults*							
Arpadi et al. [77]	Perinatally HIV-infected children, 6–16 years	59	Orally vitamin D3 100,000 IU oral every 2 months + 1 g calcium/day	Double placebo	24 months	BMD (whole body and spine)	No between-group differences before or after adjustment for stage of sexual maturation
Havens et al. [70]	HIV-infected youth on ART with or without tenofovir, 18–25 years, 55% vitamin D insufficiency or deficiency	203	Vitamin D3, 50,000 IU at 0, 4, and 8 weeks	Placebo at 0, 4, and 8 weeks	8 weeks	BTM + PTH	PTH decreased in the TDF group receiving vitamin D, not in the no-TDF group receiving vitamin D, or either placebo group, regardless of baseline 25-OHD concentration
Giacomet et al. [71]	HIV-infected children and young adults with stable disease and with vitamin D insufficiency or deficiency, 8 to 26 years	48	Orally vitamin D3 100,000 IU every 3 months	Placebo	12 months	PTH	Early (3 months) decrease in PTH, persisting at 12 months
Rovner et al. [78]	Children and young adults with HIV infection, 65% males, 86% Blacks, age 20.9 ± 3.6	58	Vitamin D3 7000 IU/day	Placebo	12 months	BMD (whole body and spine, tibia)	No significant treatment group difference
Eckard et al. [79]	HIV-infected youth 8–25 years old with vitamin D insufficiency or deficiency, 64% males, 89% Blacks, age median 20.3	102	Vitamin D3 - moderate dose 60,000 IU/month - high-dose 120,000 IU/month	Vitamin D3, standard-dose 18,000 UI/ monthly	12 months	BTM BMD (spine, hip)	- Significant decreases in P1NP and CTX in the high-dose arm only - No significant differences in BMD changes
Havens et al. [72]	Youth with HIV, RNA load < 200 copies/mL, taking TDF-containing ART for ≥ 180 days, 84% male, 74% black/African American, age 16–24	214	Daily multivitamin containing vitamin D3 400 IU and calcium 162 mg + vitamin D3 50,000 IU/month	daily multivitamin containing vitamin D3 400 IU and calcium 162 mg + Placebo	48 weeks	BMD (spine)	BMD increased in the vitamin D3 group, not in the placebo group, but without significant between-group difference
Sudjaritruk et al. [73]	Thai adolescents with perinatally acquired HIV, aged 10-20 years, on stable ART, 25OHD level median 25.5 ng/ml	200	Vitamin D3 400 IU/day + calcium 1200 mg/day + Vitamin D2 20,000 IU/ week	Vitamin D3 400 IU/day + calcium 1200 mg/day	48 weeks	BTM BMD (spine)	Greater changes in spine BMD Z-scores in high vitamin D dose versus standard-dose groups
*In adults*							
Bang et al. [80]	HIV-1-infected males, mean age 47	61	- 1 μg calcitriol and 1200 IU vitamin D3/day - 1200 IU vitamin D3/day	Placebo	16 weeks	BTM + PTH	BTM (CTX and P1NP) decreased compared to placebo in group calcitriol + cholecalciferol
Overton et al. [74]	ART-naive HIV-infected adults, 90% males	165	4000 IU/day of vitamin D3 + 500 mgx2/day calcium carbonate	Placebo	48 weeks	BMD (spine, hip)	Smaller decline in hip and spine BMD with initiation of ART
Yin et al. [81]	African American and Hispanic postmenopausal women with HIV on ART, age 56 ± 5, 74% HIV RNA ≤ 50 copies/mL	85	Vitamin D3 3000 IU/day	Vitamin D3 1000 IU/day	12 months	BTM BMD (spine, hip, radius)	No between-group differences in change in BMD, P1NP or CTX

*BMC* bone mineral content, *BMD* bone mineral density, *BTM* bone turnover markers, *PTH* parathyroid hormone, *ART* antiretroviral therapy, *25OHD* 25-hydroxyvitamin D; Vitamin D insufficiency or deficiency = serum 25-hydroxyvitamin D concentrations < 30 ng/mL

### Anti-osteoporotic Drugs

In patients with osteoporosis, bisphosphonates remain first line options in PLWH because clinical data suggest that they are well tolerated, safe, and with BMD response similar to that of the general population (Table 5). The effects of zoledronic acid persist for several years after one or two infusions [82, 83]. A single dose of zoledronic acid in non-osteoporotic, ART-naïve, HIV-infected adults initiating ART prevents the decrease of BMD [84]. A short-course of oral alendronate, started 2 weeks prior the initiation of ART and continued for a total of 14 weeks, also attenuates BMD decrease in this context [85]. There is no evidence that HIV patients are at greater risk for bisphosphonate-associated osteonecrosis of the jaw or atypical femoral fractures. For other anti-osteoporotic treatments such as denosumab or teriparatide, data are currently lacking, with only a few case reports or cohort studies [86].

**Table 5:** Interventions studies (open-label studies or placebo-controlled randomized trials) with bisphosphonates in people living with HIV

Reference	Population	Number	Duration	Intervention	Control	Endpoints	Results
*Studies with alendronate*							
Guaraldi et al. [87]	HIV-infected adults (71% men), treated with stable ART, spine or femoral neck BMD T-score < -1SD, age 45.5 ± 3.6 (intervention) 42.5 ± 3.6 (control)	41	12 months	Alendronate 70 mg/week + calcium 1000 mg/vitamin D 500 IU/day	Calcium 1000 mg/vitamin D 500 IU/day	BTM BMD (spine and hip)	Lower bone resorption (N-telopeptide) in the alendronate-treatment group compared to controls after 12 months No between-groups differences for change in BMD
Negredo et al. [88]	HIV-infected adults on stable ART, with osteoporosis age and gender unknown (older age and lower dietary calcium intake in the alendronate group)	25	96 weeks	Alendronate 70 mg/week + dietary counseling to ensure a dietary calcium intake of 1200 g/day	Dietary counseling alone	BMD (spine and hip)	BMD improved in the intervention group while decreased in the control group
Mondy et al. [89]	HIV-infected adults, males (87%), age 44 ± 1.5, on ART for ≥ 6 months, with spine BMD T-scores < -1SD	31	48 weeks	Alendronate 70 mg/week + calcium 1000 mg/vitamin D 400 IU/day	Calcium 1000 mg/vitamin D 400 IU/day	BTM BMD (spine and hip)	Greater increase of spine BMD, and decrease of bone alkaline phosphatase, osteocalcin, and urine pyridinolines and deoxypyridinolines in the alendronate group
McComsey et al. [90]	HIV-infected subjects (71% men), age median (range) 48 years (30–68, treated with stable ART, spine T-score < -1.5SD	82	48 weeks	Alendronate 70 mg/week + calcium 1000 mg/vitamin D 400 IU/day	Placebo + calcium 1000 mg/vitamin D 400 IU/day	BTM BMD (spine and hip)	Greater increase of spine and hip BMD in the alendronate group Decrease of BTM in the alendronate group
Rozenberg et al. [91]	HIV-infected (≥ 5 years or CD4 cell count nadir < 200/mm^3^) adults (95% men), with osteoporosis (spine or hip T-score ≤ -2.5SD) and CD4 cell count > 50/mm^3^, age median (range) 45 (27–75)	44	96 weeks	Alendronate 70 mg/week + calcium 500 mg/vitamin D 400 IU/day	Placebo + calcium 500 mg/vitamin D 400 IU/day	BTM BMD (spine and hip)	Greater increase of spine BMD in the alendronate group Greater decrease of alkaline phosphatase, non-significant decrease of CTX and osteocalcin
Jacobson et al. [92]; Lindsey et al. [93]	Children and adolescents (age 11–24 years), perinatally infected with HIV, on stable ART or not on ART for ≥ 12 weeks) with low spine BMD (Z score < -1.5SD)	50	96 weeks	Alendronate 70 mg/week if > 30 kg or 35 mg/week if ≤ 30 kg for 96 weeks + calcium 600–1200 mg/vitamin D 400–800 IU /day Alendronate for 48 weeks followed by placebo for 48 weeks + calcium 600–1200 mg/vitamin D 400–800 IU /day	Placebo for 48 weeks followed by alendronate for 48 weeks + calcium 600–1200 mg /vitamin D 400–800 IU /day	BMD (spine and whole body)	BMD improvement with alendronate, maintained after stopping alendronate
McGinty et al. [85]	ART-naive adults with HIV (86% male, 46% Caucasian, 34% African and 20% Hispanic), median age 35 years, initiating TDF/emtricitabine + integrase or protease inhibitors	50	50 weeks	Alendronate 70 mg/week + calcium/vitamin D3	Placebo + calcium/vitamin D3	BMD (spine and hip)	BMD loss prevented at the hip, attenuated at the spine, in alendronate group
*Studies with zoledronate*							
Bolland et al. [82, 94, 95]	HIV-infected men treated with ART for ≥ 3 months, with spine or hip BMD T-score < -0.5SD, age 49.5 ± 9.0 (intervention) 48.8 ± 9.0 (control)	43	2 years + 1 year follow-up	Zoledronate 4 mg/year (2 infusions in total) + calcium 400 mg/day + vitamin D 50,000UI/month	Placebo + calcium 400 mg/day + vitamin D 50,000UI/month	BMD (spine, hip, whole body)	Zoledronate significantly increased BMD at all sites compared to placebo Bone resorption decreased substantially by 3 months and remained stable thereafter in the intervention group No significant within‐group changes in BTM and BMD between 24 month and 5 years after the second dose
Huang et al. [96]	HIV-infected subjects (90% men), with osteopenia and osteoporosis, age 48 ± 13 (intervention) controls 49 ± 7, HIV viral load ≤ 5000 copies/ml, CD4 cell count ≥ 100 cells/μl, and stable ART (including no treatment)	30	12 months	Zoledronate 5 mg + calcium 1000 mg/vitamin D 400 IU/day	Placebo + calcium 1000 mg/vitamin D 400 IU/day	BTM BMD (spine and hip)	Increase spine and hip BMD compared to placebo Decrease of BTM in zoledronate group
Negredo et al. [83]	HIV-infected adults (87% men) on ART with low BMD (spine or hip T-score ≤ -1SD)	31	96 weeks	Zoledronate 5 mg single dose + calcium 1200–1500 mg/vitamin D 800 IU/day Zoledronate 5 mg/year for 2 years + calcium 1200–1500 mg/vitamin D 800 IU/day	Calcium 1200–1500 mg/vitamin D 800 IU/day)	BTM BMD (spine and hip)	Similar BMD increase and BTM decrease with a single dose and annual administration of zoledronate in 2 years
Ofotokun et al. [84]	Non-osteoporotic, ART-naive adults with HIV (79% men, 84% Black), initiating ART	63	48 weeks	Zoledronate 5 mg single dose	Placebo	BTM BMD (spine and hip)	65% reduction in bone resorption with zoledronate relative to the placebo arm at 24 weeks BMD loss at spine and hip prevented in zoledronate group
Hoy et al. [97]; Carr et al. [98]	HIV-infected adults (96% men) with low BMD (spine or hip T-score ≤ -1SD), TDF-treated, undetectable plasma HIV viral load	87/69	24/36 months	Continuation of TDF-based ART + zoledronate 5 mg/year for 2 years	Switch TDF to another active ART	BMD (spine and hip)	Greater increase of spine and hip BMD in the zoledronate group at 24 and 36 months

*ART* antiretroviral therapy, *BMD* bone mineral density, *BTM* bone turnover markers, *TDF* tenofovir disoproxil fumarate

### ART Regimen in Case of Bone Fragility

It has been suggested to consider or switch to “bone-friendly” ART to reduce bone loss in PLWH with established osteoporosis or multiple risk factors for developing bone fragility. TDF-sparing regimens using TAF or integrase inhibitor are also discussed in the context of renal toxicity associated with bone fragility, or renal hypophosphatemia [62, 63]. However, there is currently no data showing that initiating, or switching to a bone-protective ART regimen reduces the incidence of fracture in PLWH. The magnitude of BMD improvement is lower in patients switching from TDF to abacavir or integrase inhibitors compared to one shot of 5 mg zoledronic acid added to TDF in virologically suppressed HIV-infected adults [97, 98]. Real-world data have also indicated that combining bisphosphonates with stopping TDF results in greater improvements in BMD than stopping TDF alone [99]. Therefore, it may also be necessary to consider anti-osteoporotic drugs in case of osteoporosis or high risk of fracture, even if a switch to a bone-friendly ART regimen has been done. Another point to consider is the substantial greater bodyweight gain observed in PLWH receiving TAF, or to a lower extent an integrase inhibitor, compared to TDF [100]. Replacing TDF with TAF is also associated with weight gain, development of obesity and worsening serum lipid levels [101]. To what extent these changes of fat mass contribute to the increase of BMD or attenuation of bone loss observed with TAF or integrase inhibitors remains unexplored.

## Conclusion

HIV infection has direct and indirect effects on bone metabolism, characterized by abnormalities in bone formation and mineralization in untreated PLWH, and increased of bone resorption with initiation of ART and associated immune reconstitution. In ART-stable patients, BMD does not decrease more than in the general population, except in the presence of classical risk factors for osteoporosis, which are very common in PLWH and should be regularly assessed. Therefore, epidemiological studies have shown that the risk of fracture is higher in PLWH than in the HIV-negative population, and even higher in case of co-infection with hepatitis C. Fractures tend to occur approximately 10 years earlier in PLWH than in the general population. HIV infection itself and the type of ART regimen (especially TDF) contribute to bone loss. Reducing tenofovir plasma concentrations with TAF attenuates the decrease of BMD, but it remains unknown whether it will contribute to reduce fracture risk. Calcium and vitamin D supplementation should be considered as ART initiation, since it attenuates the decrease of BMD at this stage. In case of indication for anti-osteoporotic drug, bisphosphonates remain the reference option.
